# Association Between Physical Performance, Gait Variability, and Fall Risk in Community-Dwelling Older Adults: Predictive Validity of Step-Width Variability for Screening of Fall Risk

**DOI:** 10.3390/life15091469

**Published:** 2025-09-18

**Authors:** Yongnam Park, Youngsook Bae

**Affiliations:** 1 Department of Physical Therapy, Suwon Women’s University, Gyeonggi-do, Suwon 18333, Republic of Korea; till87@hanmail.net; 2 Department of Physical Therapy, College of Medical Science, Gachon University, 191 Hambangmoe-ro, Yeonsu-gu, Incheon 21936, Republic of Korea

**Keywords:** accidental falls, aged, gait, risk factor, physical fitness

## Abstract

**Objective**: This cross-sectional study aimed to investigate the associations between physical performance, gait variability, and fall risk in community-dwelling older adults. **Methods**: A total of 446 participants were divided into fall-risk and non-fall-risk groups. Physical performance was assessed using hand grip strength (HGS), the Timed Up-and-Go (TUG) test, and the Five Times Sit-to-Stand test (5TSTS). Spatiotemporal gait parameters and their coefficients of variation (CV) were measured on a treadmill. **Results**: Logistic regression revealed that TUG, HGS, step-width CV, and velocity CV were significantly associated with fall risk, whereas age was not. Among these, TUG and step-width CV demonstrated the highest discriminative ability (AUC = 0.708 and 0.715, respectively). **Conclusions**: Step-width CV was a particularly sensitive indicator of gait stability. These findings suggest that a combination of TUG and step-width CV may help identify older adults at risk of falls, underscoring the importance of gait variability in fall risk screening.

## 1. Introduction

Fall accidents among the elderly are on the rise, and prevention of these falls is far more important than management [1]. One in three adults over the age of 65 experiences at least one fall each year, and the incidence is even higher for those over the age of 80 [2]. Falls increase the risk of functional disability and mortality [1]. In Korea, national survey data show that 15.9–25.1% of community-dwelling older adults experience falls each year, highlighting the need for prevention strategies [3]. In older adults, osteo-sarcopenia increases the risk of falls and fractures, and fractures resulting from falls are associated with reduced life satisfaction and activities of daily living and an increased risk of death [4,5]. Therefore, early assessment of associated factors is essential for fall prevention. In older adults, poorer physical functionality is strongly and independently associated with an increased risk of falls [6]. Among the tools used to assess physical function, longer Timed Up-and-Go (TUG) test times, longer Five Times Sit-to-Stand test (5TSTS) times, and weaker hand grip strength (HGS) have been reported as factors associated with falls in community-dwelling older adults [7,8].

In the elderly, balance control is impaired by age-related declines in vestibular, proprioceptive, visual, and peripheral sensory inputs, including tactile feedback from the soles of the feet [9,10,11]. Because falls occur mainly during walking [12], many studies have evaluated the association between gait parameters during walking and fall experience. Fall experience is associated with gait parameters, with step length being a good indicator of the presence or absence of falls [13], and gait variability may be an important factor related to falls [14]. In particular, step width serves as an important indicator of mediolateral (ML) stability during walking, as an increase in step width enlarges the ML distance between the body’s center of mass and center of pressure [15], which may reflect decreased balance control [16]. Previous studies suggest that older adults have more difficulty controlling balance in the ML direction than in the anteroposterior direction, and that active control is required to control balance in the ML direction [17,18]. Gait analysis, such as gait variability, has become an important indicator for assessing human motor performance [19]. Spatial and temporal gait variability is a sensitive indicator for assessing gait function [20]. At normal walking speed, step-width variability is associated with falls over the past year in older adults [21]. Older adults exhibit greater step-width variability than younger adults [21], suggesting that lateral stability decreases with aging during walking. In addition, step-width variability and step- and stride-length variability are better than step-time variability as factors related to falls [22].

Therefore, because gait parameters such as stride length, step width, and their variability have been reported to be closely related to falls, it is important to consider both physical function and spatiotemporal gait characteristics together when evaluating fall risk. In particular, alterations in step width may reflect not only gait characteristics but also a compensatory strategy for instability in the balance control mechanism. However, the mechanisms by which these variables and their variability contribute to falls have not been clearly established. To address this gap, a comprehensive analysis that integrates physical performance tests and gait variability measures is required. Such an approach may provide valuable insights into the factors associated with falls and help improve screening strategies for older adults in the community.

The purpose of this study was to examine the associations between fall experience and physical function variables (HGS, TUG, and 5TSTS) as well as spatiotemporal gait variables (stride length, step width, cadence, and velocity) and their variability, and to identify key variables that may serve as associated factors of falls in community-dwelling older adults. We hypothesized that older adults with a history of falls would demonstrate poorer physical performance and greater gait variability than those without falls. We further hypothesized that greater step width and increased variability would be associated with risk of falls.

## 2. Materials and Methods

### 2.1. Participants and Procedures

This study was designed as a cross-sectional observational study, in which all variables were measured at a single point in time without longitudinal follow-up among community-dwelling older adults. Participants aged ≥65 years were recruited through posters and telephone interviews. The inclusion criteria were (1) individuals aged ≥65 years who were able to perform activities of daily living independently or with the aid of simple assistive devices, such as a cane; (2) those without musculoskeletal disorders (e.g., severe osteoarthritis of the hip or knee) or neurological conditions (e.g., stroke, Parkinson’s disease) that could affect current walking; and (3) those without a history of lower limb surgeries that may affect walking function (e.g., previous lower limb osteotomies, hip and knee replacements, corrective foot surgeries, lumbar spine surgery for neurological disorders), regardless of when the procedure was performed, and without any other leg surgery within the past 6 months. Those who had a Mini-Mental State Examination score of less than 21, were unable to walk independently without assistive devices, had undergone lower extremity surgery within the past 6 months, were unable to walk on a treadmill, and did not complete the measurement procedures were excluded.

Participants completed a questionnaire assessing health status, including fall history and frequency during the past six months, and underwent physical performance measurements. Spatiotemporal gait parameters and their variability were then recorded while participants walked on a treadmill. Prior to data collection, all participants performed a 3 min familiarization walk at their preferred speed. Following familiarization, a 1 min seated rest period was provided. Subsequently, participants walked on the treadmill at their preferred gait speed for 90 s, with speed adjusted as needed within ±0.1 km/h. Gait data were collected for 60 s, beginning 30 s after the onset of treadmill walking.

All data were collected in university laboratories and community centers using the same standardized protocols, equipment, and examiner training to ensure consistency across sites. Inter-rater reliability tests were conducted prior to data collection, and no systematic differences between sites were observed. The researchers were aware of the purpose of this study, but the outcome assessors were blinded. This study was conducted between 10 January 2024 and 16 August 2024. Before starting the experimental procedure, prospective participants were provided with detailed information about the study procedures and safety, and then provided written informed consent. This study was approved by the Institutional Review Board of Gachon University (1044396-202308-HR-165-01, approval date: 20 September 2023) and was conducted in accordance with the Declaration of Helsinki, and data were analyzed anonymously. The required sample size was estimated a priori using G*Power version 3.1.9.7 (Heinrich Heine University Düsseldorf, Germany), based on an expected odds ratio of 1.5, α = 0.05, and power = 0.80 for logistic regression. This indicated that a minimum of 260 participants was required. During recruitment, 479 older adults volunteered to participate. After applying the inclusion and exclusion criteria, 446 were deemed eligible and included in the final analysis. To avoid potential bias and to enhance statistical power and generalizability, all eligible participants were analyzed. The researchers were aware of this study’s objectives, whereas the outcome assessors were blinded to them.

### 2.2. Data Collection

#### 2.2.1. Physical Performance

**Figure 1:**
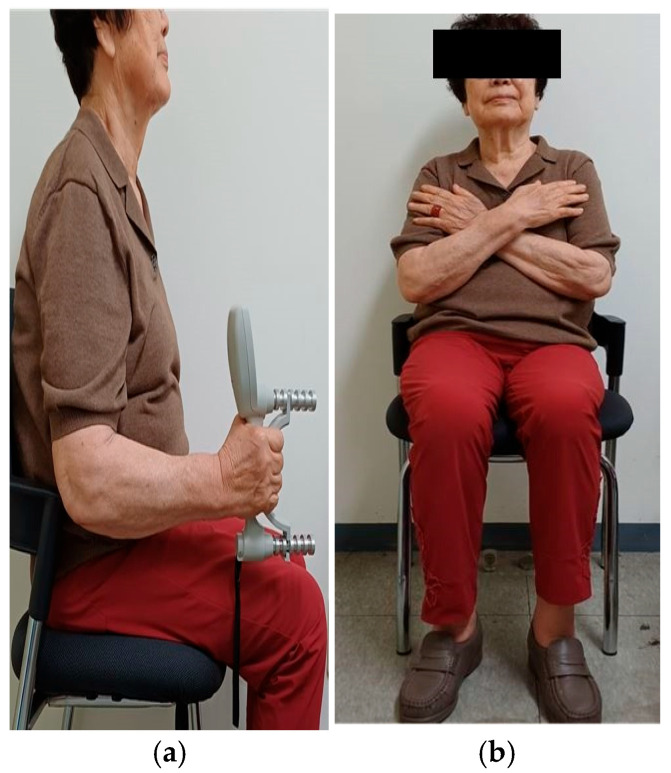
Assessment of physical performance. (**a**) Hand grip strength using dynamometer, (**b**) starting position for Five Times Sit-to-Stand test.

Physical performance measurements included HGS, the TUG test, and the 5TSTS test. HGS was assessed using a Jamar hydraulic dynamometer (Sammons Preston Inc., Bolingbrook, IL, USA). This instrument has been shown to have excellent reliability (intraclass correlation coefficient [ICC] = 0.98) in older adults [23]. Participants sat on a comfortable chair with their elbows flexed at 90°, ensuring that the upper arms were separated from the sides of the torso for accuracy, according to the standardized grip strength testing guidelines recommended by the American Society of Hand Therapists (Figure 1a). Participants were instructed to measure their dominant hand and grip the dynamometer with maximum force for 5 s. The test was conducted three times, with a 15 s rest provided between attempts. The average score from the three trials was recorded in kilograms.

To assess the TUG, participants were instructed to start in a seated position, stand up, walk 3 m to a marked area, and then return to the starting seated position. The TUG has been shown to be a highly reliable (ICC = 0.93) assessment tool for evaluating functional mobility in community-dwelling older adults [24]. The TUG test was performed twice at the participant’s normal walking speed, and the completion time was measured. The fastest time among the three trials was used, measured in seconds (s). The shortest time (s) of the two trials was recorded.

The 5TSTS involves repeating the sit-to-stand movement five times as quickly as possible. It is a valid and reliable (ICC = 0.98) measure of dynamic balance and lower limb strength in older adults [25,26]. Participants began the 5TSTS seated on a chair with a seat height of 43–45 cm. Each participant was instructed to cross their arms over their chest and sit with their back against the vertical backrest of the chair (Figure 1b) [26]. The evaluator then demonstrated how to perform the test correctly by fully extending the hips and knees to achieve a complete stand. When the evaluator said “start”, the timing began, and participants were instructed to “stand up and sit down five times as quickly as possible” without any physical assistance. The test was performed twice, with a 5 min recovery interval between tests. The average result of the two tests was calculated.

#### 2.2.2. Spatiotemporal Gait Parameter and Variability

Gait parameters and variability were assessed using the Zebris FDM-THM-S treadmill system (Zebris Medical GmbH, Isny, Germany), a reliable and sensitive device [27,28]. The treadmill platform measured 150 × 50 cm and was equipped with a capacitive pressure surface containing 7168 sensors (each approximately 0.85 × 0.85 cm). Walking speed could be finely adjusted in 0.1 km/h increments within a range of 0.2–22 km/h, and the treadmill was operated on a level surface (0% incline). The spatiotemporal gait variables analyzed in this study included stride length (cm), step width (cm), velocity (km/h), and cadence (steps/min).

The coefficient of variation (CV) is used to quantify the magnitude of variability and provides a quantitative measure of gait stability. In this study, CV was calculated by dividing the standard deviation of each parameter by its mean and expressing the value as a percentage (SD/mean × 100). Gait variability measured using CV is one of the most clinically relevant digital biomarkers for evaluating gait disorders [29]. Accordingly, gait variability can be employed as an indicator for assessing gait stability in community-dwelling older adults. Because gait variability requires at least 50 steps or 1 min of walking to ensure reliable data collection [22], gait parameters in this study were measured for approximately 60 s while participants walked on the treadmill.

### 2.3. Data Analysis

SPSS 26.0 software (IBM Corp., Armonk, NY, USA) was used for statistical analyses. Frequencies and descriptive statistics were used to assess the general characteristics of the participants. The participants were divided into two groups, the fall-experienced and non-fall-experienced groups, based on the presence or absence of a fall history. An independent sample *t*-test was used to compare the general characteristics, physical performance, gait parameters, and variability between the two groups. Prior to analysis, the assumptions of normality (Shapiro–Wilk test) and homogeneity of variance were confirmed. In addition, binary logistic regression was conducted to examine the associations between fall experience (dependent variable: 0 = non-fall, 1 = fall) and independent variables including HGS (kg), TUG (s), 5TSTS (s), and gait variability parameters (stride length CV, step width CV, cadence CV, and velocity CV), with age, sex, body weight, and height entered as covariates.

Receiver operating characteristic (ROC) curve analysis was used to evaluate the predictive performance of physical performance variables, spatiotemporal gait parameters, and variability for fall risk. An area under the curve (AUC) of 0.7–0.8 was considered acceptable, 0.8–0.9 was considered good, and ≥0.9 was considered excellent [30]. In this study, variables with an AUC of ≥0.7, indicating significant predictive power, were selected for ROC curve analysis. The cut-off values were determined using the Youden Index, which identifies the point of optimal sensitivity and specificity. All continuous data were summarized as the mean ± SD.

## 3. Results

A total of 479 older adults were recruited. However, 12 did not meet the inclusion criteria, and 21 had difficulty walking on a treadmill and could not perform the measurement procedures. Therefore, a total of 446 older adults (165 men, 281 women) were included in this study. The fall-experienced group (*n* = 145) was older than the non-fall-experienced group (*n* = 301), and physical function parameters such as HGS, TUG, the Short Physical Performance Battery, and 5TSTS were significantly lower than those in the non-fall-experienced group. Additionally, gait variables showed significantly greater cadence, stride length, step width, velocity, and cadence variability in the fall-experienced group than in the non-fall-experienced group (Table 1).

Binary logistic regression analysis for fall risk prediction revealed significant associations between physical performance variables, gait parameters, and fall risk. The 5TSTS was significantly associated with reduced fall risk (OR = 0.924, *p* = 0.016), whereas longer TUG performance time was significantly associated with increased fall risk (OR = 0.820, *p* < 0.001). In addition, step width was associated with reduced fall risk (OR = 0.934, *p* = 0.044), and among gait variability measures, only step-width CV showed a significant association with fall risk (OR = 0.881, *p* < 0.001). However, physical performance and gait variability parameters were not significantly associated with age, sex, body weight, or height (Table 2).

The AUC analysis for fall risk prediction revealed that certain physical performance and gait variables were significantly associated with fall risk. Among the physical performance variables, TUG demonstrated the highest predictive value with an AUC of 0.708 (95% CI: 0.653–0.754, *p* < 0.001), followed by 5TSTS with an AUC of 0.692 (95% CI: 0.371–0.484, *p* < 0.001). For gait parameter, step-width CV and velocity CV were significantly associated with fall risk, with AUC values of 0.715 (95% CI: 0.664–0.766, *p* < 0.001) and 0.605 (95% CI: 0.548–0.661, *p* < 0.001), respectively (Table 3).

## 4. Discussion

In this study, physical performance and gait parameters (including variability) did not show a significant association with age, indicating that fall risk is more closely associated with physical performance, gait parameters, and variability than with age. Among the physical activity levels of older adults, the HGS and 5TSTS showed significant correlations with fall experience. However, AUC analysis revealed that the 5TSTS showed a greater discriminatory power in predicting fall risk than the HGS. This suggests that, although both variables are representative indicators of muscle strength, lower extremity muscle strength is more closely related to fall risk than upper extremity muscle strength. In contrast, among physical performance variables, the TUG test was the best predictor of fall risk, with an AUC of 0.708. This finding aligns with previous studies that have identified the TUG as a key indicator for predicting functional decline, fall risk [31,32], and mobility impairment [33,34,35]. The cut-off time for the TUG to distinguish between fall risk and functional decline in community-dwelling older adults has been reported as 12.85 s and 12 s in previous studies [36,37]. In this study, the cut-off time was 12.05 s, with sensitivity of 0.593 and specificity of 0.777, which is consistent with previous research. This confirms that the TUG test can be a simple and efficient tool for assessing fall risk.

Among gait parameters and variability measures, step width and its CV were significantly associated with fall risk, suggesting that these variables are more closely related to fall risk than other gait measures. In particular, in patients with gait disorders such as Parkinson’s disease or fibromyalgia, increased step-width variability may reflect gait instability and impaired motor coordination [38,39], which may increase the risk of falls. This highlights the importance of analyzing gait pattern variability, as it may provide deeper insight into subtle instabilities that are not captured by gait parameters such as stride length or speed, and supports the results of previous studies showing that gait variability is closely related to fall risk [40,41].

In particular, step-width CV demonstrated significant discriminatory power with an AUC of 0.715, a sensitivity of 0.421, and a specificity of 0.877. Lateral stability during walking is controlled through active adjustment of lateral foot position, and by controlling lateral foot placement, walking stability can be maintained. At normal walking speeds, foot placement is strictly regulated; however, this control is substantially reduced at slower speeds [42]. This indicates that impaired stability is accompanied by difficulties in foot placement control. An increase in step-width variability suggests challenges in balance control during walking, which may be closely related to unstable gait patterns, a major cause of falls. The results of our study support previous reports that increased step-width variability is associated with decreased balance ability and an increased risk of falls [21,22].

This demonstrates that step-width CV is an important indicator of stability and balance during walking [43]. The cut-off value for step-width CV was calculated to be 21.7%. This value could be used as a criterion to distinguish between fall risk and non-fall-risk. As the TUG test alone has limitations for identifying individuals at high risk of falling among community-dwelling older adults [44], the authors suggest that combining the TUG test and stride CV would be more effective in predicting fall risk in community-dwelling older adults.

This study has some limitations. (1) This study was based on a single measurement and did not include long-term monitoring. Therefore, the findings should be interpreted with caution, and future research should incorporate longitudinal assessments to better capture changes in gait and fall risk over time. (2) Limited generalizability: This study was conducted in community-dwelling older adults, which limits the generalizability of the results to other populations, such as younger age groups, older adults residing in care facilities, and those with chronic diseases. Future studies should recruit participants from more diverse populations, including institutionalized older adults or those with chronic diseases, to improve the generalizability of our findings. (3) Laboratory conditions: The measurements of physical function and gait variables were based on laboratory conditions and short-term assessments, which did not account for real-life walking environments or long-term gait changes. Since gait was assessed under short-term laboratory conditions, future research should incorporate long-term monitoring using wearable devices in real-world settings. (4) Complex interactions not considered: The complex interactions between factors such as muscle strength, balance ability, neurological function in fall risk, previous pathologies, and the intake of medications with neurological effects (from anxiolytics to sleep modulators, analgesics, anti-inflammatories, etc.) were not fully explored. Future studies should consider multifactorial models including muscle strength, neurological functions, comorbidities, and medication use to better understand the complex mechanisms underlying fall risk. (5) Limited definition of fall risk: Fall risk was defined solely on the basis of a history of falls in the last 6 months, without considering other possible causes or more detailed risk classifications. Future studies should utilize comprehensive fall risk assessments that include prospective monitoring. (6) A significant age difference (*p* = 0.016) was observed between the fall and non-fall groups. Binary logistic regression analysis including age as a covariate showed no significant effect on the results, indicating that the findings remained valid. However, future studies are needed to further validate these results in groups without age differences. Additionally, although this study did not include systematic follow-up interventions, future studies should incorporate these prevention strategies to strengthen the clinical applicability and practical validity of gait-based fall risk assessment.

Despite these limitations, this study possesses several important strengths. First, while previous studies have primarily used gait speed or stride length as indicators of fall risk, this study found that stride-length variability was a particularly sensitive indicator for detecting gait instability in community-dwelling older adults. Furthermore, by presenting a threshold of 21.7% for stride-length variability, this study provides a practical basis for early diagnosis of older adults at high risk for falls and for developing clinical guidelines for fall prevention. Second, we comprehensively analyzed various physical functions, including the HGS, TUG, 5TSTS, gait indices, and variability to comprehensively assess their association with fall risk. Third, we used gait variability indices, specifically the step-width CV, to predict fall risk, which proved to be a more sensitive indicator than existing measures of gait variability. Finally, because this study was conducted on community-dwelling older adults, our findings provide valuable baseline data for developing fall risk prediction models and prevention strategies applicable to real-world settings.

## 5. Conclusions

This study demonstrated that physical function and gait variability are associated with fall risk, with TUG and step-width CV showing particularly significant associations. Therefore, evaluating TUG and step-width CV may provide a more accurate assessment of fall risk. These findings offer fundamental data for the early identification of high-risk groups and the development of tailored fall prevention programs.

## Figures and Tables

**Table 1:** General characteristics, physical performance, and spatiotemporal gait parameter and variability of participants.

	Fall-Experienced Group (*n* = 145)	Non-Fall-Experienced Group (*n* = 301)	t/X^2^	*p*
Sex (male/female)	47 (32.5)/98 (67.6)	118 (39.2)/183 (60.8)	1.935	0.164
Age (years)	79.64 ± 8.07	77.90± 6.63	2.416	0.016
Height (cm)	155.53 ± 8.10	157.32 ± 11.93	−1.637	0.102
Weight (Kg)	59.27 ± 9.19	61.38 ± 9.80	−2.786	0.031
K-MMSE (score)	25.39 ± 3.92	26.00 ± 3.27	−1.737	0.083
Fall frequency	1.50 ± 1.07	0	24.193	<0.001
HGS (kg)	20.61 ± 7.71	22.68 ± 8.32	−2.517	0.012
TUG (s)	13.41 ± 4.37	10.54 ± 2.56	8.673	<0.001
5TSTS (s)	14.90 ± 5.16	11.81 ± 3.60	7.308	<0.001
Stride length (cm)	73.63 ± 25.23	76.28 ± 23.40	−1.092	0.275
CV (%)	7.33 ± 6.19	6.41 ± 6.14	1.471	0.142
Step width (cm)	11.50 ± 3.43	11.35 ± 3.44	0.432	0.666
CV (%)	21.84 ± 10.85	14.35 ± 6.04	9.337	<0.001
Velocity (km/h)	2.43 ± 0.94	2.57 ± 0.88	−1.505	0.133
CV (%)	8.04 ± 5.86	6.49 ± 6.56	2.418	0.016
Cadence (step/min)	110.57 ± 18.85	112.57 ± 18.06	−1.076	0.283
CV (%)	4.84 ± 3.94	4.34 ± 4.84	1.072	0.284

K-MMSE: Korean Mini-Mental State Examination, HGS: hand grip strength, TUG: Timed Up-and-Go test, 5TSTS: Five Times Sit-to-Stand, CV: coefficient of variation.

**Table 2:** Binary logistic regression analysis for fall-risk prediction based on physical performance and gait parameters and variability.

Variable	B	Odds Ratio (OR)	95% CI	*p*-Value
Physical performance				
Age	0.004	1.004	0.973–1.004	0.794
Sex	−0.235	0.791	0.422–1.481	0.463
Height	−0.008	0.992	0.961–1.024	0.625
Weight	0.024	1.024	0.995–1.054	0.101
HGS (kg)	−0.011	0.989	0.948–1.032	0.625
TUG (s)	−0.199	0.820	0.751–0.895	<0.001
5TSTS (s)	−0.079	0.924	0.869–0.983	0.016
Gait parameter and variability				
Age	−0.032	0.968	0.931–1.007	0.106
Sex	−0.003	0.997	0.575–1.729	0.991
Height	−0.006	0.994	0.969–1.020	0.660
Weight	0.024	1.024	0.997–1.053	0.086
Stride length (cm)	0.000	1.000	0.945–1.059	0.990
CV (%)	0.026	1.026	0.965–1.091	0.413
Step width (cm)	−0.068	0.934	0.874–0.998	0.044
CV (%)	−0.127	0.881	0.853–0.910	<0.001
Velocity (km/h)	0.022	0.954	0.197–5.298	0.979
CV (%)	−0.047	1.005	0.897–1.016	0.141
Cadence (step/min)	−0.002	0.998	0.984–1.012	0.768
CV (%)	0.041	1.042	0.973–1.116	0.240

HGS: hand grip strength, TUG: Timed Up-and-Go test, 5TSTS: Five Times Sit-to-Stand, CV: coefficient of variation.

**Table 3:** AUC analysis of physical performance and gait parameters for fall risk prediction.

Variable	AUC	95% CI	*p*-Value
Physical performance			
Grip strength	0.428	0.638–0.745	0.012
TUG (s)	0.708	0.653–0.754	<0.001
5TSTS (s)	0.692	0.371–0.484	<0.001
Gait parameter and variability			
Stride length (cm)	0.477	0.418–0.535	0.430
CV (%)	0.552	0.495–0.609	0.072
Step width (cm)	0.514	0.457–0.572	0.625
CV (%)	0.715	0.664–0.766	<0.001
Velocity (km/h)	0.457	0.399–0.515	0.145
CV (%)	0.605	0.548–0.661	<0.001
Cadence (step/min)	0.469	0.411–0.527	0.298
CV (%)	0.573	0.517–0.630	0.010

TUG: Timed Up-and-Go test, 5TSTS: Five5 Times Sit- to- Stand, CV: coefficient of variation.

## Data Availability

The data presented in this study are available on request from the corresponding author due to privacy or ethical restrictions.
